# Sensing Approaches Exploiting Molecularly Imprinted Nanoparticles and Lossy Mode Resonance in Polymer Optical Fibers

**DOI:** 10.3390/nano13162361

**Published:** 2023-08-18

**Authors:** Francesco Arcadio, Laurent Noël, Domenico Del Prete, Mimimorena Seggio, Luigi Zeni, Alessandra Maria Bossi, Olivier Soppera, Nunzio Cennamo

**Affiliations:** 1 Department of Engineering, University of Campania Luigi Vanvitelli, Via Roma 29, 81031 Aversa, Italy; francesco.arcadio@unicampania.it (F.A.); domenico.delprete@unicampania.it (D.D.P.); luigi.zeni@unicampania.it (L.Z.); 2 CNRS, IS2M UMR 7361, Université de Haute-Alsace, 68100 Mulhouse, France; laurent.noel@uha.fr; 3 Université de Strasbourg, 67000 Strasbourg, France; 4 Department of Biotechnology, University of Verona, Strada Le Grazie 15, 37134 Verona, Italy; mimimorena.seggio@univr.it

**Keywords:** lossy mode resonance (LMR), plastic optical fibers (POFs), metal oxides (MOs), LMR sensors, molecularly imprinted nanoparticles (nanoMIPs), human transferrin (HTR)

## Abstract

In this work, two different lossy mode resonance (LMR) platforms based on plastic optical fibers (POFs) are developed and tested in a biochemical sensing scenario. The LMR platforms are based on the combination of two metal oxides (MOs), i.e., zirconium oxide (ZrO_2_) and titanium oxide (TiO_2_), and deposited on the exposed core of D-shaped POF chips. More specifically, two experimental sensor configurations were obtained by swapping the mutual position of the Mos films over to the core of the D-shaped POF probe. The POF–LMR sensors were first characterized as refractometers, proving the bulk sensitivities. Then, both the POF–LMR platforms were functionalized using molecularly imprinted nanoparticles (nanoMIPs) specific for human transferrin (HTR) in order to carry out binding tests. The achieved results report a bulk sensitivity equal to about 148 nm/RIU in the best sensor configuration, namely the POF-TiO_2_-ZrO_2_. In contrast, both optical configurations combined with nanoMIPs showed an ultra-low detection limit (fM), demonstrating excellent efficiency of the used receptor (nanoMIPs) and paving the way to disposable POF–LMR biochemical sensors that are easy-to-use, low-cost, and highly sensitive.

## 1. Introduction

Lossy mode resonance (LMR) phenomenon can be triggered when the interaction between an incident light and a nanometric film of a specific material takes place. As well described in the scientific literature, LMR can be excited when the real part of the thin film permittivity is positive and higher in magnitude than its own imaginary part and the one of the external surrounding medium [1]. More specifically, this phenomenon is triggered for a certain value of the thin film thickness when the lossy modes approach the cut-off. The results of this interaction consist of attenuation dips observable in the transmitted spectra [1,2]. Typically, for this purpose, thin films of metal-oxides (MOs) and high refractive index polymers are used to excite LMR [3].

Having the excellent properties related to the utilization of optical fibers as sensors, LMR was frequently associated with these waveguides (made in silica and plastic) in the development of groundbreaking sensors [4,5,6,7,8,9]. The first example of LMR sensors based on optical fiber was proposed by del Villar et al. [10], who demonstrated that optimizing the thickness of an indium tin oxide (ITO) coating deposited onto a uncladded optical fiber makes it is possible to observe multiple LMR resonance wavelengths. By exploiting diverse metal oxide layers, the same research group presented many interesting experimental configurations based on this technique [11,12,13,14,15,16]. 

Given the excellent results achievable with LMR optical fiber-based refractometers, in a later stage, these kinds of platforms were effectively coupled with several kinds of receptors to develop biochemical sensors. For instance, Socorro et al. [17] developed an immunoglobulin G biosensor by depositing a PAH/PSS layer-by-layer thin film on an unclad optical fiber. Śmietana et al. [18] recently proposed a device based on an ITO overlaid section of a multimode fused silica core optical fiber for the label-free biosensing of avidin. Furthermore, Usha et al. [19] coupled a molecularly imprinted polymer (MIP) with an LMR probe based on a layer of zinc oxide and molybdenum disulfide (ZnO/MoS_2_) deposited over the unclad core of an optical fiber for urinary p-cresol diagnosis. 

Recently, Cennamo et al. presented the proof of concept of a sensing platform based on D-shaped plastic optical fibers (POFs) capable of triggering the LMR phenomenon together with the surface plasmon resonance (SPR) one [20]. It is important to underline that the LMR phenomenon can be excited by the interaction between incident light and an ultra-thin layer of specific materials, whereas the surface plasmon resonance (SPR) can be triggered at the interface between an ultra-thin metal film and a dielectric medium. The materials in SPR used to trigger the resonance phenomenon are limited to noble metals (such as gold, silver, etc.). However, a wider range of materials with peculiar optical properties (such as metal oxides, high refractive index polymers, etc.) can excite the LMR phenomenon [21]. In [20], the proposed probe exploits the combination of two metal oxides, i.e., zirconium oxide (ZrO_2_) and titanium oxide (TiO_2_), deposited on a modified POF. In [20], a gold nanofilm covers the MOs on the top. In this method, it was possible to observe a multi-resonance phenomenon due to the simultaneous excitation of SPR and LMR [20]. These sensing platforms were recently coupled to molecularly imprinted nanoparticles (nanoMIPs) to conduct binding tests, comparing the obtained performance with the state-of-the-art SPR and LMR biochemical sensors [22].

This work developed different optical probes consisting of a ZrO_2_ and TiO_2_ combination bilayer deposited on the core of a D-shaped POF sensitive area. These kinds of platforms, unlike the ones extensively presented in [20,22], do not include the upper gold nanofilm in such a way as to exploit only the LMR phenomenon. These sensor configurations were experimentally tested for their binding behavior by coupling them with the nanoMIPs layer. More specifically, two different sensor configurations obtained by swapping the relative positions of the metal oxide coatings were realized, and both sensors were experimentally characterized. 

The presented optical platforms, based on D-shaped POFs coupled with two different metal oxides (TiO_2_ and ZrO_2_) bilayer combinations, were thoroughly studied from an optical point of view in [20], where a gold nanofilm is deposited over the MOs. In [20], four sensor configurations based on metal oxide monolayers and bilayers were optically characterized and compared. The optical platforms based on the bilayers (TiO_2_-ZrO_2_ and ZrO_2_-TiO_2_), which showed appropriate LMR performance in bulk sensitivity [20], were explored to evaluate the binding sensitivity using nanoMIPs as a recognition element. In this work, the nanoMIPs layer is in contact with MOs directly, without the gold nanofilm used in [22]. Currently, to the best of our knowledge, the LMR technique is infrequently implemented with nanoMIPs. In particular, by exploiting nanoMIPs, the binding sensitivity improves thanks to the high value of receptor efficiency [22,23,24]. In fact, when the NanoMIPs-analyte binding occurs, the capability to convert variations of analyte concentration into refractive index changes is ultra-efficiency [22,23,24].

Generally, this kind of synthetic receptor is extensively used in different plasmonic-based sensors [23,24,25]. MIPs are considered one of the most promising potential alternatives to antibodies (ABs) in the field of molecular recognition elements [26]. Indeed, MIPs technology can overcome the drawbacks of ABs because they are inexpensive, easy to fabricate, and adapted to various molecules. MIPs are prepared to recognize a wide variety of different compounds in terms of dimension and properties, such as proteins, drugs, and pollutants [26,27,28].

Along this line of argument, the proposed LMR platforms, exploiting MOs [29] on D-shaped POFs, were functionalized with a nanoMIPs receptor layer specific for the human transferrin (HTR) in order to carry out binding measurements. Selectivity tests were also performed on both the realized sensor configurations.

## 2. Materials and Methods 

### 2.1. Chemicals

Methacrylic acid (99%) (MAA), titanium (IV) isopropoxide (97%) and zirconium (IV) propoxide in n-propanol (70 wt.%) were purchased from Merck and n-propanol from Alfa Aesar and were used as received. Acrylamide (Aam), 1-ethyl-3-(-3-dimethylaminopropyl) carbodiimide hydrochloride (EDC), N-hydroxysuccinimide (NHS), Lysil-lysine (Lys-Lys), methacrylic acid (MAA), N-tert-butylacrylamide (TBAm), N,N′-methylene bisacrylamide (BIS), N,N,N′,N′-tetramethyl ethylenediamine (TEMED), ammonium persulfate (APS), sodium dodecyl sulfate (SDS), Tris (hydroxymethyl)-aminomethane (TRIS), sodium dihydrogen phosphate, disodium monohydrogen phosphate, hydrochloric acid, sodium hydroxide, sodium chloride, Tween-20, 3-Aminopropyl)Triethoxysilane, acetonitrile, acetic acid, ethanol, were from Sigma–Aldrich (Darmstadt, Germany). The proteins human serum transferrin (HTR), horseradish peroxidase (HRP), trypsin were from Sigma–Aldrich (Darmstadt, Germany). A 40% *w*/*w* APS stock solution was freshly prepared prior to polymerization. 

### 2.2. Synthesis of nanoMIPs 

Molecularly imprinted nanoparticles (nanoMIPs) were synthesized according to [30]. A total monomer concentration of 0.1% (*w*/*v*) was used in the syntheses. Aam, MAA, TBAm were added at 8, 8, and 4% (mol/mol) respectively, together with 80% (mol/mol) of BIS in phosphate buffer (PB) pH 7.4 supplemented with SDS 0.01% (*w*/*v*). The template (HTR) was added at a concentration of 1.2 μM. The mixture was sonicated for 5 min and bubbled with N_2_ for 30 min. The initiators, APS (0.04% *w*/*v*) and TEMED (0.03% *w*/*v*), were added and the polymerization was carried out at 20 °C for 20 h. At the completion of the polymerization, the pH was adjusted to 8 with 50 mM Trizma-base and Trypsin was added to the mixture in a 1:25 (*w*/*w*) ratio with respect to the template for 2 h at 30 °C, in order to remove the template. Finally, the nanoparticles (nanoMIPs) were extensively washed (3 L of MilliQ water) using a Vivaflow 50 system (100,000 MWCO) (Sartorius Stedim, Firenze, Italy) and lyophilized.

### 2.3. Experimental Setup

A cost-effective and simple-to-use setup was used to test the POF–LMR sensors. It consists of a halogen lamp as a white light source (HL–2000–LL, manufactured by Ocean Optics, Orlando, FL, USA) and a spectrometer (FLAME-S-VIS-NIR-ES, Ocean Optics, Orlando, FL, USA) to collect the transmitted light through the LMR sensor. In particular, the LMR sensor was placed between the source and the spectrometer, and SMA connectors were used to connect all setup components with POF. In the end, the spectrometer was connected to a laptop to process the experimental data.

### 2.4. Optical Characterization

The optical performances for both LMR platforms were tested with water-glycerin mixtures with a variable refractive index (RI) ranging from 1.332 (water) to 1.385.

LMR spectra at different RI were obtained by normalization on the spectrum acquired with air as an external medium, where the resonance condition is not satisfied for both experimental sensor configurations. In these types of sensors, the resonance wavelength ($\lambda_{res}$) shifts by changing the refractive index of the external medium deposited upon the sensitive area. So, the bulk sensitivity $S_{b}$ can be defined as

(1) $$S_{b}=\frac{\delta \lambda_{res}}{\delta n}\left[\frac{nm}{RIU}\right]$$

where δn is the variation in refractive index that caused a shift in resonance wavelength of $\delta \lambda_{res}$. The error bars for both configurations were calculated as the maximum measured variation of the resonance wavelength and resulted equal to 0.2 nm. 

The LMR resonance wavelength variations (∆λ) at different RI were obtained with respect to the water (1.332 RIU). In order to estimate the bulk sensitivity, the LMR resonance wavelength variation (∆λ) is plotted versus the refractive index of the external medium, and a linear fitting of the experimental values was performed. 

### 2.5. HTR Detection: Binding Measurement Protocol

The functionalized LMR platforms were tested for the binding of the target analyte, HTR, by dropping alternatively on the platform 80 μL of analyte solutions in the concentration range 17 fM–280 fM. After 5 min of incubation, a PBS washing step was carried out, and the spectra were acquired using the blank solution (PBS) as a bulk solution. Data were fitted to the Langmuir model equation considering an averaged single binding site per nanoMIPs particle. The shift in LMR resonance wavelength, calculated with respect to the blank (i.e., PBS without the analyte), versus the HTR concentration was fitted to the Langmuir model reported in Equation (2).

(2) $$\left|∆\lambda \right|=\left|\lambda_{c}-\lambda_{0}\right|=\left|\Delta \lambda_{max}\right|\cdot \left(\frac{c}{K+c}\right)$$

where $\lambda_{c}$ is the resonance wavelength at the analyte concentration *c*, $\lambda_{0}$ is the resonance wavelength value at the blank, $\Delta \lambda_{max}$ is the maximum value of ∆λ (calculated by the saturation value minus the blank value) and K is a dissociation constant. The error bars were calculated as the maximum variation in resonance wavelength, resulting equal to about 0.2 nm. It is essential to underline that although the full width at half maximum (FWHM) of the LMR peak is not comparable, for instance, to the narrower SPR obtained by monomodal waveguides. The LMR resonance wavelength value can be well determined within an uncertainty of ±0.2 nm (error bars) exploiting the high sensitivity with respect to the monomodal waveguides.

The Langmuir curves, used to fit the experimental data, were obtained using Origin Pro 9 software (Origin Lab. Corp., Northampton, MA, USA).

## 3. LMR-Based HTR Sensors: POF–LMR Platforms and nanoMIPs

### 3.1. POF–LMR Sensors Fabrication 

The TiO_2_ and ZrO_2_ MO thin films were prepared following the previously published protocol [29]. This protocol relies on depositing sol-gel solutions followed by Deep-UV laser curing. Two solutions were prepared: one containing titanium oxo-clusters (TiOCs) and another containing zirconium oxo-clusters (ZrOCs). The metal precursors were first added in 2 mL of MAA, and then 2 mL of n-propanol was added after 5 min of stirring. Before adding deionized water, the mixture was stirred for 10 min. The resulting formulations have molar ratios of 1:8:20 for Ti:MAA:DI and 1:10:22 for Zr:MAA:DI. After adding water, the solutions were stirred for 1 h and underwent an ageing time of 1 day before the dilution step. A specific volume of n-propanol was added in order to reach a given dilution rate.

The fabrication process of the POF–LMR platforms can be summarized as follows. Firstly, a POF (with a core of PMMA and 1 mm in total diameter) is embedded into a resin block and modified using a lapping procedure in order to obtain the D-shaped POF area [20]. The D-shaped POF region has a length of 1 cm and a width of about 600 μm. The last step consists of the deposition of the metal oxides prepared as reported above (ZrO_2_ and TiO_2_) on the exposed core of the fiber through a Deep-UV-curing based process [20].

More specifically, two different sensor configurations were deployed: the first one, named “Design 1”, consists of a bilayer TiO_2_-ZrO_2_ formed by a TiO_2_ nanolayer deposited upon the modified POF and a ZrO_2_ layer on the top of the latter; the second one, named “Design 2”, consists of a bilayer ZrO_2_-TiO_2_, similar to the latter but obtained by swapping the position of the metal oxides. The concentration of the solutions was adapted to obtain 40 ± 2 nm for each layer. In both configurations, the total thickness of the metal oxide bilayer is equal to about 80 ± 2 nm. The cross-linking of the metal oxide layer was achieved by Deep-UV irradiation using a 193 nm ArF excimer laser (Excistar from Coherent). After laser curing, the refractive indexes of each layer were 1.63 (ZrO_2_) and 1.79 (TiO_2_). More details on the deposition process are reported in [20].

### 3.2. POF-LMR Functionalization Process

**Figure 1:**
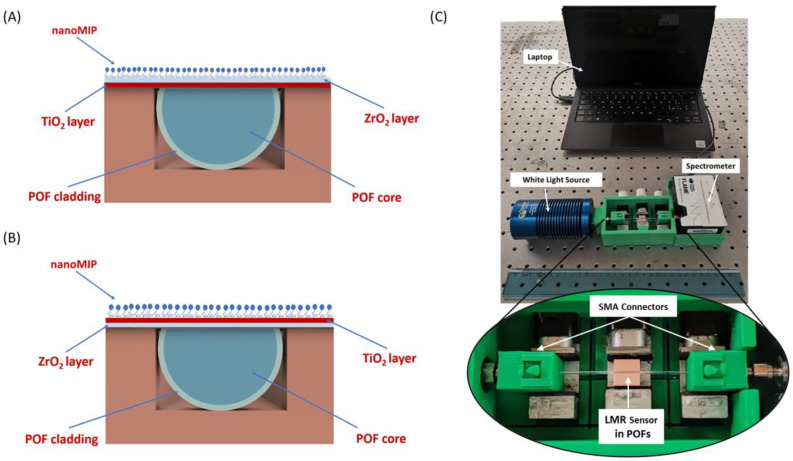
Outline of the cross-sections of (**A**) Design 1 (POF-TiO_2_-ZrO_2_) and (**B**) Design 2 (POF-ZrO_2_-TiO_2_), both combined with the same nanoMIPs layer. (**C**) Experimental setup used to test both the POF–LMR–nanoMIPs sensors; Zoom: detail of the platform holder.

For the functionalization process, the POF–LMR platforms were salinized in a water solution at 10% *w*/*v* of (3-Aminopropyl)Triethoxysilane for 3 h, followed by rinsing and nanoMIPs (0.3 mg/mL) were coupled with NHS (12.5 mM) and EDC (12.5 mM) in 50 mM MES buffer pH 5.5 for 2 h. Platforms were then treated with 2 mM ethanolamine to quench unreacted species. Figure 1A,B report an outline of both the POF–LMR designs functionalized with the same nanoMIPs layer. An actual image of the experimental setup used to test both sensor configurations is shown in Figure 1C.

## 4. Results

### 4.1. Design 1 (POF-TiO_2_-ZrO_2_)

#### 4.1.1. Bulk Sensitivity for Design 1 (POF-TiO_2_-ZrO_2_)

**Figure 2:**
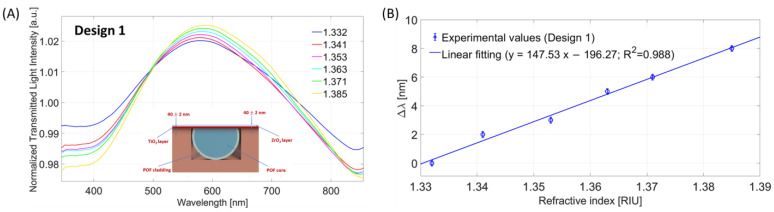
Bulk sensitivity test for Design 1: (**A**) LMR spectra (transmitted spectra normalized on the reference spectrum) at different external medium refractive indices. (**B**) LMR resonance wavelength variations, calculated with respect to the water (RI = 1.332), versus the refractive index together with the linear fitting of the experimental values.

At first, the bulk sensitivity for Design 1 (POF-TiO_2_-ZrO_2_) was evaluated by monitoring the resonance wavelength variations induced by the changes in the solution’s refractive index in contact with the sensitive layer. Figure 2A shows the normalized transmitted spectra for increasing RI of the water–glycerin solutions ranging between 1.332 and 1.385. As shown in Figure 2A, the LMR wavelength increases when increasing the external RI (redshift). Figure 2B reports the linear fitting of the obtained experimental values. It is possible to estimate the LMR bulk sensitivity as the slope of the linear fitting function using Equation (1), resulting in an equal to about 148 nm/RIU (see Figure 2B).

#### 4.1.2. Binding Sensitivity for Design 1 (POF-TiO_2_-ZrO_2_-nanoMIPs)

**Figure 3:**
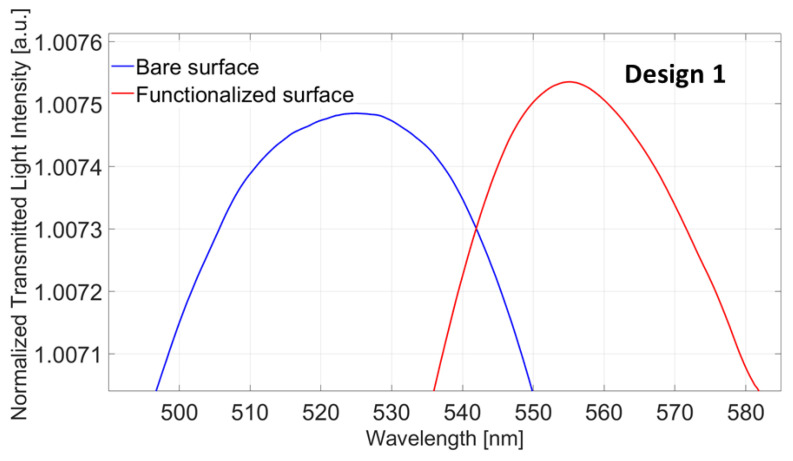
LMR spectra acquired with water as an external medium before (blue line) and after (red line) the functionalization process.

The first analysis, with regards to the functionalized Design 1 (POF-TiO_2_-ZrO_2_-nanoMIPs), was carried out to verify the effectiveness of the nanoMIPs immobilization process. Figure 3 shows the LMR spectra before (blue line) and after (red line) the functionalization step. A clear red shift in the LMR resonance wavelength of about 30 nm can be seen. This result confirms the immobilization of the nanoMIPs layer upon the platform’s sensitive surface. In particular, when the nanoMIPs layer is present on the sensitive surface, the measured refractive index increases with the same bulk solution (water).

After confirming the effectiveness of the immobilization process, the performance of the functionalized Design 1 platform at the HTR-binding was tested according to the binding measurement protocol reported in Section 2.5.

**Figure 4:**
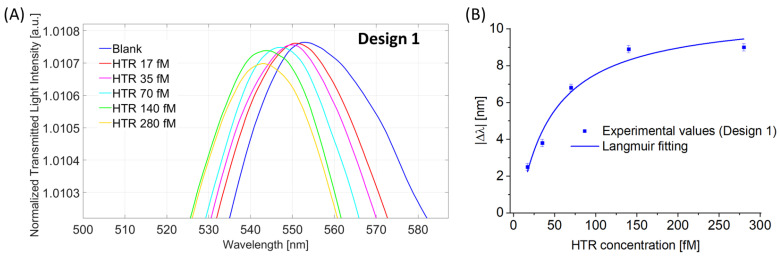
Binding test for Design 1. (**A**) LMR spectra at different HTR concentrations in the range from 17 fM to 280 fM. (**B**) Absolute value of the shift in LMR resonance wavelength (|∆λ|), calculated with respect to the blank versus HTR concentrations, together with Langmuir fitting of the experimental values and error bars.

Figure 4A reports the normalized transmitted spectra (LMR spectra) obtained with different HTR concentrations ranging from 17 fM to 280 fM. From the above figure, it is possible to observe that the LMR resonance wavelength decreases (blue shift) at increasing HTR concentrations. This blue shift is a well-known phenomenon when dealing with nanoMIPs since it was extensively demonstrated that the analyte-nanoMIPs interaction causes a shrinkage of the nanoparticles, reducing the measured refractive index at the sensitive surface [22,23,24,30]. 

The experimental data were fitted to the Langmuir model (Equation (2)) that describes the binding interaction between the analyte (HTR) and the receptor layer (nanoMIPs), as shown in Figure 4B. The parameters of the Langmuir curve data are reported in Table 1.

**Figure 5:**
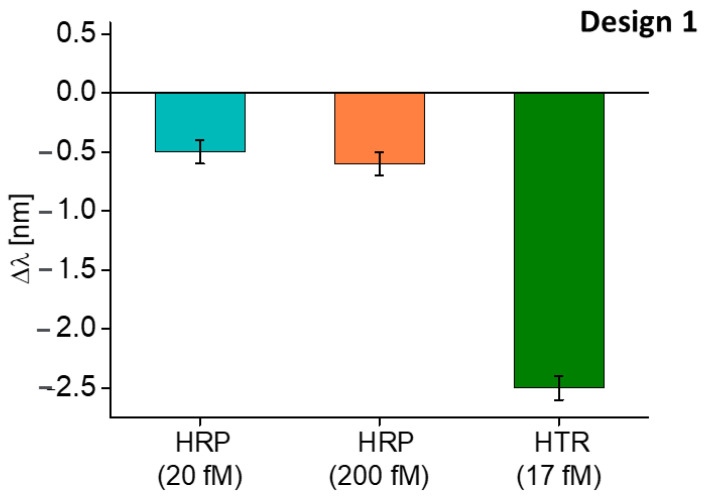
Selectivity test of Design 1: comparison between the shift in resonance wavelength caused by the interferent (HRP) at different concentrations and the analyte (HTR).

Moreover, a selectivity test with an interferent at different concentrations was performed. For this test, the horseradish peroxidase (HRP) was chosen, and it was tested at two different concentrations, namely 20 fM and 200 fM. The shift in the resonance wavelength obtained with HRP was compared with the one obtained in HTR-binding. As reported in Figure 5, the shifts produced by the interferent (HRP) are negligible with respect to the one produced by the analyte (HTR), even if the interferent concentrations (HRP) are greater than the analyte concentration (HTR).

### 4.2. Design 2 (POF-ZrO_2_-TiO_2_)

#### 4.2.1. Bulk Sensitivity for Design 2 (POF-ZrO_2_-TiO_2_)

**Figure 6:**
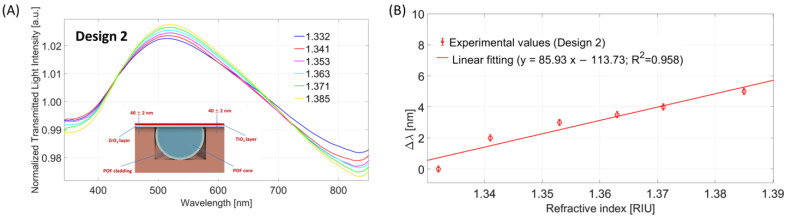
Bulk sensitivity test for Design 2: (**A**) LMR spectra at different external medium refractive indices; (**B**) LMR resonance wavelength variations, calculated with respect to the water, versus refractive index together with linear fitting of the experimental values and error bars.

In a similar way as adopted for the optical characterization of Design 1, Design 2 (POF-ZrO_2_-TiO_2_) was characterized using the same water–glycerine solutions. The experimental results are reported in Figure 6. As shown in Figure 6A, also in this case, the LMR wavelength is red-shifted when the external RI increases.

From the linear fitting function of the experimental data reported in Figure 6B, an LMR bulk sensitivity equal to about 86 nm/RIU was obtained.

#### 4.2.2. Binding Sensitivity for Design 2 (POF-ZrO_2_-TiO_2_-nanoMIPs)

**Figure 7:**
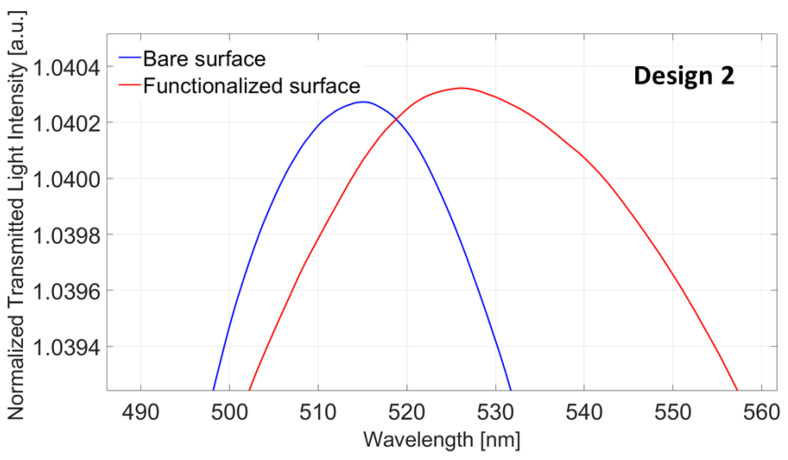
Design 2: LMR spectra acquired with water as an external medium before (blue line) and after (red line) the functionalization process.

As for Design 1, the same analysis was carried out on functionalized Design 2 (POF-ZrO_2_-TiO_2_-nanoMIPs) to verify the efficacy of the functionalization process. Figure 7 shows the normalized transmitted spectra (the LMR spectra) before (blue line) and after (red line) the functionalization process when the water is used as a bulk solution. It shows a clear red shift in the LMR resonance wavelength of about 15 nm, confirming the nanoMIPs layer deposition. As it can be observed, in this case, the resonance wavelength variation is smaller than the one obtained for Design 1, since Design 2 showed a lower bulk sensitivity from the previous optical characterization.

**Figure 8:**
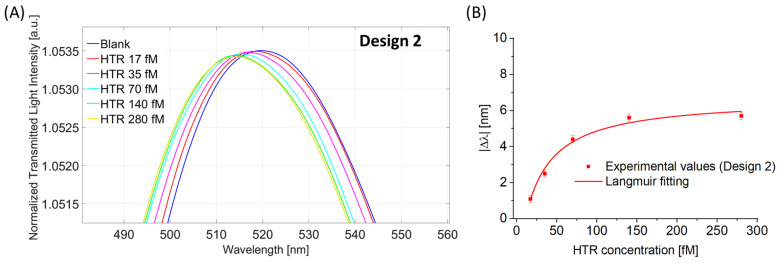
Binding test for Design 2. (**A**) LMR spectra at different HTR concentrations in the range from 17 fM to 280 fM. (**B**) Absolute value of the shift in LMR resonance wavelength (|∆λ|), calculated with respect to the blank, versus HTR concentration together with Langmuir fitting of the experimental values and error bars.

Analogously to what was reported for Design 1, the HTR-binding test was performed in Design 2. Figure 8A shows the LMR spectra acquired with HTR solutions in the same concentration range (17 fM to 280 fM). Also in this case, it is possible to observe that the LMR resonance wavelength decreases (blue shift) for an increment of the HTR concentration (when the binding occurs, the RI of the nanoMIPs decreases). Figure 8B shows the absolute value of the variation in LMR resonance wavelength, calculated with respect to the blank, versus the HTR concentration with the Langmuir fitting of the experimental values (see Equation (2)) and the error bars.

Table 2 shows the parameters of the Langmuir curve used to fit the experimental data reported in Figure 8B.

**Figure 9:**
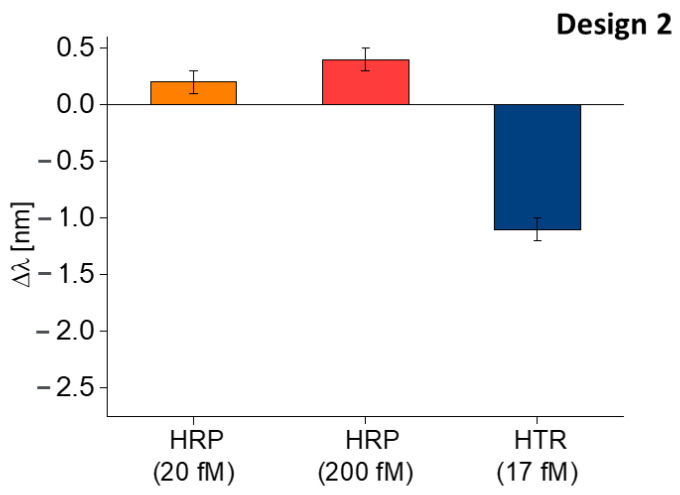
Selectivity test of Design 2: comparison between the shift in resonance wavelength caused by the interferent (HRP) at different concentrations and the analyte (HTR).

Finally, the HRP, in the same modality adopted for Design 1 was used to test the sensor’s selectivity. From Figure 9, the shift in resonance wavelength caused by the interferent (HRP) appears negligible compared to the shift caused by the analyte (HTR). Moreover, in this case, the HRP produced a slight red shift in the resonance wavelength, attributed to a not specific interaction caused by the deposition of the substance upon the sensitive surface.

## 5. Discussion

**Figure 10:**
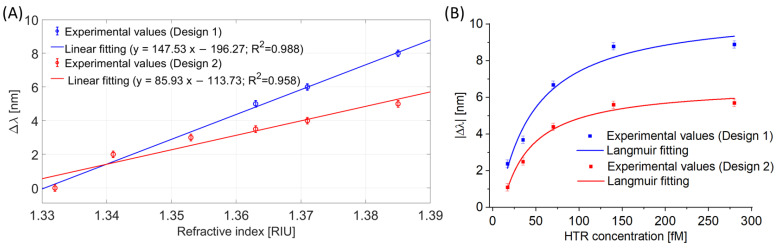
(**A**) LMR resonance wavelength variations, calculated with respect to the water, versus the refractive index and linear fittings for both sensor configurations. (**B**) Experimental variation of the LMR resonance wavelength versus HTR concentration, along with Langmuir fittings of the experimental values, for both configurations.

In order to compare the two experimental designs, Figure 10 shows for Design 1 (POF-TiO_2_-ZrO_2_) and Design 2 (POF-ZrO_2_-TiO_2_) the LMR bulk sensitivities (Figure 10A) and the binding isotherms obtained via the same nanoMIPs layer (Figure 10B).

As shown in Figure 10A, Design 1 denoted a higher LMR bulk sensitivity with respect to Design 2 (≅148 nm/RIU and ≅86 nm/RIU, respectively). These results confirm the one obtained in [20].

Figure 10B shows the experimental values and the Langmuir fittings for the analyzed sensor configurations.

By using the experimental values reported in the previous analysis (Table 1 and Table 2) and the Langmuir model reported in Equation (2), when considering c much lower than *K*, i.e., at low analyte concentration, Equation (2) can be considered linear, and the slope is called the “sensitivity at low concentrations” (*S_lc_*):

(3) $$S_{lc}=\frac{\left|\Delta \lambda_{max}\right|}{K}$$

The limit of detection (*LOD*) can be calculated as the ratio between three times the standard deviation of the blank (St. error of $\lambda_{0}$) and the sensitivity at low concentrations:

(4) $$LOD=\frac{3\times St.error(\lambda_{0})}{S_{lc}}$$

In the end, it can be possible to define the affinity constant as follows:

*K*_*aff*_ = 1/*K* (5)

Table 3 reports the above-defined parameters calculated for both HTR sensor configurations.

From these parameters, it is possible to observe that the performance at binding for both sensor designs appears quite similar. The performances obtained by the LMR–nanoMIPs sensors presented in this work were comparable to those obtained for SPR–LMR–nanoMIPs sensors recently reported in [22]. Nevertheless, the optical platforms presented here could be preferred since they do not foresee any sputtering procedure (for gold deposition), thus making the fabrication process faster and simpler.

Moreover, in Table 4, the proposed HTR sensors (nanoMIPs combined with POF-TiO_2_-ZrO_2_ and POF-ZrO_2_-TiO_2_) were compared with other LMR platforms functionalized with different kinds of receptors. It is possible to confirm that the proposed biochemical sensors show a lower LOD with respect to all the other LMR sensors already reported in the literature, specifically for other proteins having a similar molecular weight to HTR. 

As reported in [24], the high binding sensitivity is related to the receptor efficiency, i.e., the capability to convert variations of analyte concentration into refractive index changes. In this work, the adopted receptor (nanoMIPs), which denoted a high efficiency because of its peculiar characteristic to deform at binding, was allowed to reach LODs in the femtomolar range [22,23,24]. 

## 6. Conclusions

In this work, two LMR-based sensor configurations based on POF platforms were designed, realized, and optically characterized by swapping the order of two metal oxides, i.e., zirconium oxide and titanium oxide. Moreover, innovative chemical sensors were realized; coupling the produced optical platforms with nanoMIPs, we were able to recognize the HTR. The so obtained optical–chemical sensors were tested to detect ultra-low concentrations of HTR in the femtomolar range. The performance at binding appears quite similar for both experimental sensor configurations, thanks to the properties of the nanoMIPs. However, the best-performing design, consisting of Design 1 (POF-TiO_2_-ZrO_2_-nanoMIPs), denoted a LOD equal to about 4.5 fM and a sensitivity at low concentrations of about 0.3 nm/fM. Moreover, the key aspect of the work is demonstrating the high efficiency of the receptor layer (nanoMIPs) can be used to obtain femtomolar range in the detection of analytes, even in the case of the worst optical probe.

In conclusion, the coupling between LMR–POF platforms and nanoMIPs layers led to the development of innovative HTR sensors with an ultra-low limit of detection (LOD). It is also important to underline that the proposed sensing approach could be used for other substances by changing the nanoMIPs imprinting.

## Figures and Tables

**Table 1:** Design 1: parameters of the Langmuir curve used to fit the experimental values (see Figure 4B).

λ_0_ [nm]		Δ*λ_max_* [nm]		K [fM]		Statistics	
Value	St. Error	Value	St. Error	Value	St. Error	Χ^2^	R^2^
−1.855	0.453	10.899	1.351	35.918	28.672	13.676	0.937

**Table 2:** Design 2: parameters of the Langmuir curve used to fit the experimental values (see Figure 8B).

λ_0_ [nm]		Δ*λ_max_* [nm]		K [fM]		Statistics	
Value	St. Error	Value	St. Error	Value	St. Error	Χ^2^	R^2^
−1.265	0.785	6.692	0.542	22.631	14.493	0.128	0.968

**Table 3:** Sensor’s chemical parameters for HTR detection.

Configuration	$S_{lc}$ [nm/fM]	LOD [fM]	$K_{aff}$ [fM^−1^]
Design 1 (POF-TiO_2_-ZrO_2_-nanoMIPs)	0.30	4.48	0.028
Design 2 (POF-ZrO_2_-TiO_2_-nanoMIPs)	0.29	7.96	0.044

**Table 4:** Comparison between several LMR-based sensor configurations developed for different analytes.

Sensor Configuration	Receptor	Analyte	LOD	Reference
ITO overlayer on multimode optical fiber fused silica core	amine group	avidin	0.15 [nM]	[18]
ITO films on D-shaped optical fiber	aptamer	C-reactive protein (CRP)	6.20 [nM]	[31]
Planar waveguide coated with a titanium dioxide (TiO_2_) thin-film	antibody	anti-IgG	10 [nM]	[32]
ITO thin layer on uncladded multimode fiber	aptamer	thrombin	100 [nM]	[33]
Design 1 (POF-TiO_2_-ZrO_2_)	nanoMIPs	HTR	4.48 [fM]	This work
Design 2 (POF- ZrO_2_-TiO_2_)	nanoMIPs	HTR	7.96 [fM]	This work

## Data Availability

The data presented in this study are available on request from the corresponding author.
